# Proteomic Identification of Phosphorylation-Dependent Septin 7 Interactors that Drive Dendritic Spine Formation

**DOI:** 10.3389/fcell.2022.836746

**Published:** 2022-05-04

**Authors:** Sujin Byeon, Bailey Werner, Reilly Falter, Kristian Davidsen, Calvin Snyder, Shao-En Ong, Smita Yadav

**Affiliations:** ^1^ Graduate Program in Neuroscience, University of Washington, Seattle, WA, United States; ^2^ Department of Pharmacology, University of Washington, Seattle, WA, United States; ^3^ Human Biology Division, Fred Hutchinson Cancer Research Center, Seattle, WA, United States

**Keywords:** dendritic spines, septin, 14-3-3, phosphoregulation, proteomics

## Abstract

Septins are a family of cytoskeletal proteins that regulate several important aspects of neuronal development. Septin 7 (Sept7) is enriched at the base of dendritic spines in excitatory neurons and mediates both spine formation and spine and synapse maturation. Phosphorylation at a conserved C-terminal tail residue of Sept7 mediates its translocation into the dendritic spine head to allow spine and synapse maturation. The mechanistic basis for postsynaptic stability and compartmentalization conferred by phosphorylated Sept7, however, is unclear. We report herein the proteomic identification of Sept7 phosphorylation-dependent neuronal interactors. Using Sept7 C-terminal phosphopeptide pulldown and biochemical assays, we show that the 14-3-3 family of proteins specifically interacts with Sept7 when phosphorylated at the T426 residue. Biochemically, we validate the interaction between Sept7 and 14-3-3 isoform gamma and show that 14-3-3 gamma is also enriched in the mature dendritic spine head. Furthermore, we demonstrate that interaction of phosphorylated Sept7 with 14-3-3 protects it from dephosphorylation, as expression of a 14-3-3 antagonist significantly decreases phosphorylated Sept7 in neurons. This study identifies 14-3-3 proteins as an important physiological regulator of Sept7 function in neuronal development.

## Introduction

Septins are evolutionarily conserved cytoskeletal proteins important for diverse cellular processes including cell division, regulation of actin and microtubule dynamics, localization of scaffolding proteins, and membrane trafficking (Mostowy and Cossart, 2012). Septins structurally contain a GTP-binding domain and a variable N- and C-terminal domain, which oligomerize with each other to form symmetric filaments and higher order structures such as rings (Sirajuddin et al., 2007; Mendonça et al., 2021). In humans, there are 13 septin genes within four homology groups: *SEPT2 (SEPT1, SEPT2, SEPT4,* and *SEPT5*), *SEPT3* (*SEPT3, SEPT9,* and *SEPT12*), *SEPT6* (*SEPT6, SEPT8, SEPT10, SEPT11*, and *SEPT14*), and *SEPT7* (*SEPT7*) (Kinoshita, 2003a). Within these septin oligomers, septins from the same group are interchangeable, suggesting potential redundancy in their function. Notably, forming its own group, Septin7 (Sept7) is a unique and non-redundant core component of the septin complexes (Kinoshita, 2003b).

Several members of the septin family were found to be enriched in postsynaptic density (PSD) fractions in the mouse brain, by a mass spectrometry analysis, with Sept7 suggested to be the most abundant (Walikonis et al., 2000). Sept7 is expressed throughout all stages of neuronal differentiation and localizes at axonal and dendritic branching points as well as at the base of dendritic protrusions (Tada et al., 2007; Xie et al., 2007). Depletion of Sept7 leads to decreased branching of axon and dendrites both *in vitro* and *in vivo* (Tada et al., 2007; Xie et al., 2007; Hu et al., 2012; Ageta-Ishihara et al., 2013). Moreover, an increase in the number of immature dendritic filopodia was observed when Sept7 was knocked down (Tada et al., 2007). Sept7 localizes at the base of dendritic spines to create an important diffusion barrier for membrane protein entry into the dendritic spine head (Ewers et al., 2014). Furthermore, Sept7 was found to be a phosphorylation target of thousand-and-one amino acid kinase 2 (TAOK2), a serine/threonine kinase encoded by the autism risk gene *TAOK2* (de Anda et al., 2012; Yadav et al., 2017; Richter et al., 2018; Nourbakhsh et al., 2021). Phosphorylation of Sept7 by *TAOK2* at an evolutionarily conserved C-terminal tail residue T426 was shown to be essential for spine maturation. While in its unphosphorylated state Sept7 localizes to the base of dendritic spines and filopodia, when phosphorylated at T426, Sept7 relocates to the dendritic spine head (Yadav et al., 2017). Although mechanisms through which phosphorylation induces Sept7 translocation are unclear, it was found that preventing this phosphorylation leads to increased dendritic filopodia as well as mislocalization of synaptic scaffold proteins to the dendritic shaft instead of the spine. This eventually led to formation of mislocalized synapses on the dendritic shafts (Yadav et al., 2017). How phosphorylation at the conserved T426 residue on the C-terminal tail of Sept7 regulates its function is unknown. The C-terminal tail extends perpendicularly out to the axis of the oligomeric septin filament (Sirajuddin et al., 2007). This is thought to facilitate protein–protein interactions in addition to lateral associations between septin filaments (Marques et al., 2012; Finnigan et al., 2015; Finnigan et al., 2016).

Herein, we show that phosphorylation of Sept7 C-terminal tail at the T426 residue can be mediated by several members of the TAO kinase family, including TAOK1 and two distinct isoforms of TAOK2 kinase. Furthermore, Sept7 phosphorylation at T426 was found to increase during development in rat embryonic hippocampal neurons. Sept7 phosphorylation was found to be important for spine maturation, as expression of phosphomimetic Sept7 T426D in early stages of neuronal development (DIV9) leads to precocious maturation of dendritic spines. Using live confocal imaging, we show that phosphorylated Sept7 is stably associated with the dendritic spine head over time in contrast with the wild-type and phosphomutant Sept7. To identify potential mechanisms through which phosphorylated Sept7 contributes to dendritic spine maturation, we performed an unbiased proteomic screening to identify phosphorylation-dependent binding partners of Sept7. Among the identified candidate interactors were several isoforms of 14-3-3 proteins and actin-binding proteins. Since 14-3-3 proteins bind and modulate functions of phosphorylated proteins, we tested whether phosphorylated Sept7 associates with 14-3-3 proteins. Using biochemical assays, we demonstrate that phosphorylated Sept7 interacts with the 14-3-3 gamma (14-3-3γ) isoform. Furthermore, we found that 14-3-3γ is enriched in dendritic spines compared to other 14-3-3 isoforms. Finally, we found that disrupting 14-3-3 and Sept7 interaction perturbs the level of phosphorylated Sept7 and maturation of dendritic spines in neurons, revealing an important role of this interaction in regulating neuronal development.

## Materials and Methods

### Antibodies and Plasmids

Antibodies used for these experiments include: anti-HA-tag (Mouse, ProteinTech, Thermo Fisher Scientific, 50–173–6,449), phospho-TAOK2 (S181) (Rabbit, R&D Systems, PPS037), GFP (Mouse, Roche, 11,814,460,001), GST (Mouse, Invitrogen, MA4-004), 14-3-3 pan isoform (Rabbit, CST, #8312), and 14-3-3 gamma (Mouse, Sigma, MA1-16587). Rabbit phospho-Septin7 (pT426) antibody was generated, as described previously (Yadav et al., 2017). Human TAOK2α was PCR amplified from pCMV-Sp6-TAOK2 plasmid (Ultanir et al., 2014) and cloned into sfGFP-C1 vector (Addgene #54579) using restriction sites HindIII and MfeI. Human TAOK2β cDNA was obtained from Transomics (#BC152413) and cloned into the sfGFP-C1 vector using the same restriction sites. Similarly, TAOK1 cDNA from Transomics (#BC144067) was inserted into the sfGFP-C1 vector using restriction enzymes. Addgene plasmids used for these experiments include: HA-14-3-3 zeta (#116888) and pcDNA3-HA-14-3-3 gamma (#13274). EYFP-Difopein and EYFP-Control were gifts from Dr. Yi Zhou (Florida State University).

### Immunoprecipitation Kinase Assays

HEK293T cells were grown in Dulbecco’s modified Eagle medium (DMEM) containing 10% fetal bovine serum (FBS) and 1% penicillin–streptomycin (Thermo Fisher Scientific). Expression constructs sfGFP-tagged TAOK1, TAOK2α, and TAOK2β (3 μg) were transfected with JetOptimus, following the manufacturer’s instructions. After 24 h post-transfection, cells were collected and incubated in HKT lysis buffer (25 mM HEPES pH 7.2, 150mM KCl, 1% Triton X-100, 2 mM DTT, and 1X protease inhibitor (Roche, cOmplete™ EDTA free)) for 30 min on ice prior to homogenization with a 25-gauge syringe needle. Pierce™ Protein G Agarose beads (Thermo Fisher Scientific) were washed with HKT buffer thrice. The supernatant was collected from cell lysates through centrifugation at 800g for 5 min, precleared with Protein G Agarose beads for 30 min, and immunoprecipitated with the GFP antibody overnight at 4°C. Beads were washed with HKT buffer twice, incubated with high salt HKT buffer (25 mM HEPES pH 7.2, 1M NaCl, 1% Triton X-100, 2 mM DTT, and 1X protease inhibitor (Roche, cOmplete™ EDTA free)) for 10 min, and washed with HK buffer (25 mM HEPES, 150mM KCl, 2 mM DTT, and 1X protease inhibitor (Roche, cOmplete™ EDTA free)) twice and then with kinase buffer (20 mM Tris HCl pH 7.5, 10 mM MgCl2, 1 mM DTT, 1X protease inhibitor (Roche, cOmplete™ EDTA free)) twice at 4°C. Kinase assay was performed by incubating the beads with the kinase buffer, 1 mM ATP, and 10X phosphatase inhibitor (Thermo Fisher Scientific, Halt) at 30°C for 45 min at 920 rpm. To assay for phosphorylation of Septin7 by TAO kinases, purified GST-tagged Sept7 C-terminal tail (321–438 amino acids) was also added to the reaction. Samples were prepared by adding NuPAGE™ LDS sample buffer (Thermo Fisher Scientific) containing 125 mM DTT, heating for 10 min at 95°C, and centrifuging at 5,000g for 5 min. Samples were run on NuPAGE™ 4–12% bis-tris polyacrylamide gels (Thermo Fisher Scientific) with NuPAGE™ MOPS running buffer (Thermo Fisher Scientific) at 165 V for 20 min and then at 175 V for 50 min. Gels were transferred to Immobilon-P membrane at 100 V for 1 h. The blots were blocked with 5% BSA blocking buffer and probed with phospho-TAO2 (S181) and phospho-Sept7 (T426) antibodies at 1:500 dilution overnight at 4°C followed by 3 h incubation with HRP-conjugated secondary antibody at 1:5,000 dilution at room temperature. The kinase activity was quantified by normalizing phospho-TAOK2 signal intensity to that of the GFP signal and phospho-Sept7 signal intensity to that of the GST signal.

### Protein Purification

Septin 7 C-terminal tail (321–438 amino acids) was cloned into pGEX4T1 vector using restriction sites BamHI and XhoI and transformed into the BL21 *E. coli* bacterial strain to bacterially express GST-tagged Sept7C-WT (Yadav et al., 2017). A 25 ml starter culture grown from a single colony overnight was used to inoculate 1 L culture, which was allowed to grow at 37°C until OD600 reached 0.6. Protein expression was induced by adding IPTG at the final concentration of 0.3 mM and growing the culture for additional 5 h at 30°C. Cells were harvested by centrifugation at 4500 g for 15 min at 4°C, washed with ice-cold PBS, and then resuspended in lysis buffer (50 mM Tris pH 8.0, 5 mM EDTA, 150 mM NaCl, 10% glycerol, 5 mM DTT, 1X protease inhibitor (Roche, cOmplete™ EDTA free), and 2 mM PMSF). To further lyse the cells, they were incubated with 4 mg of lysozyme on ice for 30 min followed by addition of 0.5% Triton X-100 and sonication. The supernatant was collected after a 30-min spin at 25,000 g and incubated with prewashed GST beads (Thermo Fisher Scientific) for 2 h. Beads were washed with wash buffer (PBS + 1 mM DTT +0.1% Tween 20) and then with wash buffer without detergent. Bound protein were eluted and collected in fractions by glutathione elution buffer at pH 8.0 (50 mM Tris pH 8.0, 250 mM KCl, 1 mM DTT, and 25 mM glutathione).

### Co-Immunoprecipitation

Co-immunoprecipitation assays were performed in HEK293T cells grown in DMEM media (Thermo Fisher Scientific, Gibco) with 10% fetal bovine serum (Axenia) and 1% penicillin–streptomycin (Invitrogen). Cells were grown to confluence in a flat-bottom plate with 35-mm deep wells at 5% CO_2_ and 37°C and transfected with 2.0 μg of HA-14-3-3 gamma, HA-14-3-3 zeta, or 1.5 μg of both gamma and zeta using JetOptimus, following the manufacturer’s instructions. Cells were co-transfected with 1.0 μg of GFP-tagged Sept7-WT. Approximately 36 h post-transfection, cells were treated with 0.1 μM okadaic acid for 10 min and lysed with 1 ml HKT buffer (25 mM HEPES pH7.2, 100 mM KCl, 1% Triton X-100, 1 mM DTT, 1 mM EDTA, phosphatase inhibitor (Thermo Fisher Scientific, Halt), and protease inhibitor (Roche, cOmplete™ EDTA free)) per 2-well construct. Lysate was incubated on ice for 20 min prior to homogenization with a 25-gauge syringe needle. Homogenate was pelleted via centrifugation at 6,000 g and 4°C for 5 min. Pierce™ Protein G Agarose (Thermo Fisher Scientific) beads and EZview™ Red Anti-HA Affinity Gel (Millipore-Sigma) beads were washed twice for 5 min at 4°C and re-suspended in 150 μL HKT per sample tube. Pellet supernatant was pre-cleared with 20 μL Pierce™ Protein G Agarose (Thermo Fisher Scientific) beads, and pre-cleared supernatant was collected for input samples. Remaining volume of pre-cleared supernatant was immunoprecipitated with 20 μL EZview™ Red Anti-HA Affinity Gel (Millipore-Sigma) beads. Beads were washed three times with HKT and twice with HK buffer (25 mM HEPES pH 7.2, 100mM KCl, 1 mM DTT, and 1 mM EDTA). Both immunoprecipitation and input samples were prepared with 4X LDS sample buffer (Thermo Fisher Scientific) and 125 mM DTT followed by heat treatment at 95°C for 10 min. Samples were centrifuged at 14,000g for 5 min at 4°C and electrophoresed on NuPAGE™ 4–12% bis-tris polyacrylamide gels (Thermo Fisher Scientific) with NuPAGE™ MOPS running buffer (Thermo Fisher Scientific) for 40 min at 160 V.

Neuronal lysates were prepared at DIV18 with HKT buffer (25 mM HEPES pH7.2, 100 mM KCl, 1% Triton X-100, 1 mM DTT, 1 mM EDTA, and protease inhibitor (Roche, cOmplete). Lysates were precleared with agarose beads and then incubated with 14-3-3 gamma (mouse) or 14-3-3 pan (Rabbit)–bound sepharose beads overnight. Beads were washed thrice with HKT buffer and twice with buffer without detergent. In experiments where phosphospecific interaction was probed, Protein G beads with immunoprecipitated 14-3-3γ were incubated overnight with neuronal lysates that were prepared in 500 μL of lysis buffer (50 mM HEPES, 100 mM NaCl, 2 mM DTT, 1% TritonX100, and 1 mM MnCl_2_) and pretreated with or without 10 μL of lambda protein phosphatase (NEB, Cat#P0753) at 30°C for an hour. Beads were then washed thrice with lysis buffer and twice with buffer without detergent before running on SDS-PAGE gels.

### Western Blot

For Western blot analysis, gels were transferred to Immobilon-P PVDF membrane (Millipore-Sigma) with transfer buffer (25 mM Tris, 192 mM glycine, and 20% (v/v) Methanol) for 60 min at 100 V. Blots were cut below the 50 kDa mark to produce one half-membrane consisting of high molecular weights to detect phosphorylated Septin 7 and one half-membrane consisting of low molecular weights to detect 14-3-3 isoforms (endogenous or HA-tagged). Blots were blocked in 5% milk (5% milk powder (Carnation), 50 mM Tris-Cl, 150 mM NaCl, and 0.1% Tween™ 20 (Thermo Fisher Scientific)) or 2% BSA (2% bovine serum albumin (VWR), 50 mM Tris-Cl, 150 mM NaCl, and 0.1% Tween™ 20 (Thermo Fisher Scientific)) blocking buffer for 1 hour at room temperature and incubated in primary antibody overnight at 4°C. Primary rabbit antibody to detect phosphorylated Septin 7 (T426) was diluted to 1:250 and applied to high molecular weight half-membrane, and primary mouse antibody to detect HA-tag or endogenous 14-3-3 was diluted to 1:1,000 and applied to low molecular weight half-membrane. Blots were washed with blocking buffer at room temperature before secondary antibody incubation. Blots were incubated in HRP-conjugated secondary antibody at 1:1,000 dilution for 3 hours at room temperature. Blots were washed successively with blocking buffer, TBST (50 mM Tris-Cl, 150 mM NaCl, and 0.1% Tween™ 20 (Thermo Fisher Scientific)), and TBS (50 mM and 150 mM NaCl). Western blot images were visualized with Pierce™ ECL Western Blotting Substrate (Thermo Fisher Scientific) and captured with the ChemiDoc Imager (Bio-Rad).

### Peptide Pulldown and Mass Spectrometry

Synthetic peptide corresponding to Sept7 C-terminal tail residues 416–438 were commercially synthesized with an N-terminal cysteine residue and T426 in either phosphorylated or unphosphorylated forms (Elim Biopharmaceuticals). Peptides were coupled to SulfoLink beads (Invitrogen), according to manufacturer’s protocol. In brief, 0.5 mg of peptide was dissolved in 1 ml of coupling buffer (50 mM Tris and 5 mM EDTA, pH8.5) and then TCEP was added to a final concentration of 25 mM. Beads (50 ul for each peptide) were washed four times in the coupling buffer, quenched with l-cysteine-HCl and then incubated with 100 ul of 0.5 mg/ml P15 mouse brain lysate. Beads were washed four times with lysis buffer, containing 20 mM Tris pH 7.5, 100 mM NaCl, 10 mM MgCl2, 0.5 mM DTT, 1% Triton X-100, 0.1% deoxycholic acid, and protease inhibitors (Roche), and thrice with lysis buffer without detergent. Proteins bound to beads were then denatured by adding 3x bead volumes of 8M urea/10 mM Tris pH 8.0 to resuspend beads. TCEP was added to a final concentration of 1 mM and incubated at room temperature for 30 min with thermomixer/slow vortex. Chloroacetamide (CAM) was added to final concentration of 3 mM and incubated at room temperature for 10 min with thermomixer/slow vortex. TCEP was added to quench excess CAM after alkylation is complete. The pH was then adjusted to 8. For protein digestion, LysC (Mass Spec Grade) at 1:100 enzyme:substrate ratio was added and incubated at 37°C for 2 h on a thermomixer with gentle agitation. Then 3x reaction volumes of TEAB was added to dilute urea to less than or equal to 2M urea. Then pH was adjusted to 8.0. Trypsin (MS grade, Promega) was added at 1:100 enzyme:substrate ratio and incubated at 37°C for 12–16 h with gentle agitation. Digestion was stopped by adding TFA to a final concentration of 1%. Peptide samples were desalted on C18 StageTips. Peptide samples were separated on an Thermo Dionex UltiMate 3000 RSLCnano System (Thermo Fisher Scientific) using 20-cm long fused silica capillary columns (100 µm ID, laser pulled in-house with Sutter P-2000, Novato CA) packed with 3 μm 120 Å reversed phase C18 beads (Dr. Maisch, Ammerbuch, DE). Liquid chromatography (LC) solvent A was 0.1% (v/v) aq. Acetic acid and LC solvent B was 20% of 0.1% (v/v) acetic acid and 80% acetonitrile. The LC gradient was 100-min long with 5–35% B at 300 nL/min. MS data was collected using an Orbitrap Elite mass spectrometer (Thermo Fisher Scientific).

### Mass Spectrometry Data Analyses

Data-dependent analysis was applied using top15 selection with CID fragmentation. Data raw files were analyzed by MaxQuant/Andromeda (Cox et al., 2011) version 1.6.2.8 using protein, peptide, and site FDRs of 0.01, a score minimum of 40 for modified peptides and 0 for unmodified peptides, and a delta score minimum of 17 for modified peptides and 0 for unmodified peptides. MS/MS spectra were searched against the UniProt mouse database (updated July 2016). MaxQuant search parameters are as follows: variable modifications included Oxidation (M) and Phospho (S/T/Y). Carbamidomethyl (C) was a fixed modification. Maximum missed cleavages were 2, enzyme was trypsin/P, and maximum charge was 7. The initial search tolerance for FTMS scans was 20 ppm and 0.5 Da for ITMS MS/MS scans. Label-free quantification (LFQ) of MS intensities from five process replicates/condition was processed with Perseus. Protein groups were filtered to remove proteins ‘only identified by site’ as well as reverse and potential contaminants. MS intensities were log2 transformed, and missing values were imputed by randomly selecting from a distribution downshifted by 1.8 and a width of 0.3. Significance was calculated by a two-sample Student’s t-test.

MS raw data (ID MSV000088699) is publicly available at repository UCSD Massive database and can be accessed freely at https://massive.ucsd.edu.

### Immunofluorescence

Rat embryonic hippocampal neurons were grown on coverslips coated with 0.1M borate buffer (50 mM boric acid and 12.5 mM sodium tetraborate in water, pH 8.5), 60 ug/mL poly-d-Lysine (Sigma), and 2.5 ug/mL laminin (Sigma) in 12-well plates. For immunofluorescence, neurons were fixed with warm 4% paraformaldehyde + 4% sucrose in PBS, followed by 60-min incubation in blocking buffer (0.2% Triton X-100, 10% normal goat serum (Jackson Labs), and 0.2M glycine pH 7.2) at room temperature. Primary antibody was incubated overnight at 4°C followed by secondary antibody for 3 hours at room temperature. Coverslips were mounted on slides using Fluoromount-G.

### Neuronal Culture

Hippocampi were obtained from E17–E18 Sprague Dawley rat embryos (Envigo), trypsin dissociated, and plated at a density of 150,000 cells per 18 mm coverslip (Fisher Scientific) and 50,000–500,000 cells per 35 mm glass bottom dish (MatTek). Dishes were coated as previously described. Neurons were seeded in plating media (10% fetal bovine serum heat inactivated, 20% dextrose, 1x Glutamax (Invitrogen), 1x penicillin/streptomycin (Thermo Fisher Scientific), Eagle’s MEM with Earle’s BBS (Lonza)) for 4 hours. Media was changed to maintenance media (B27 (Invitrogen), 1x penicillin/streptomycin, 1x Glutamax, and neurobasal media (Invitrogen)). Half of the media was replaced with new maintenance media every 3–4 days. Transfection of mammalian expression constructs in neurons was performed using Lipofectamine 2000, following the manufacturer’s instructions. Neurons were either fixed for 48 h after transfection using 4% PFA+ 4% sucrose, or imaged live 48 h after transfection.

### Microscopy and Image Analyses

All live and fixed cell imaging was performed using a Nikon Ti2 Eclipse-CSU-X1 confocal spinning disk microscope equipped with four laser lines 405, 488, 561, and 670 nm and a sCMOS Andor camera for imaging. The microscope was caged within the OkoLab environmental control setup enabling temperature and CO_2_ control during live imaging. Imaging was performed using Nikon 1.49 ×100 Apo, ×60, or ×40 oil objectives. All image analyses were done using the open access Fiji software.

### Statistics

All statistics except for the mass spectrometry data were performed in GraphPad software Prism 9.0. Two group comparisons were made using unpaired *t*-test, unless otherwise stated. Statistically, p-value less than 0.05 was considered significant. All experiments were done in triplicate, unless stated otherwise, and experimental sample size and p-values are indicated with the corresponding figures.

## Results

### Conserved C-Terminal Tail in Septin 7 is Phosphorylated by TAO Kinases During Neuronal Development

**FIGURE 1:**
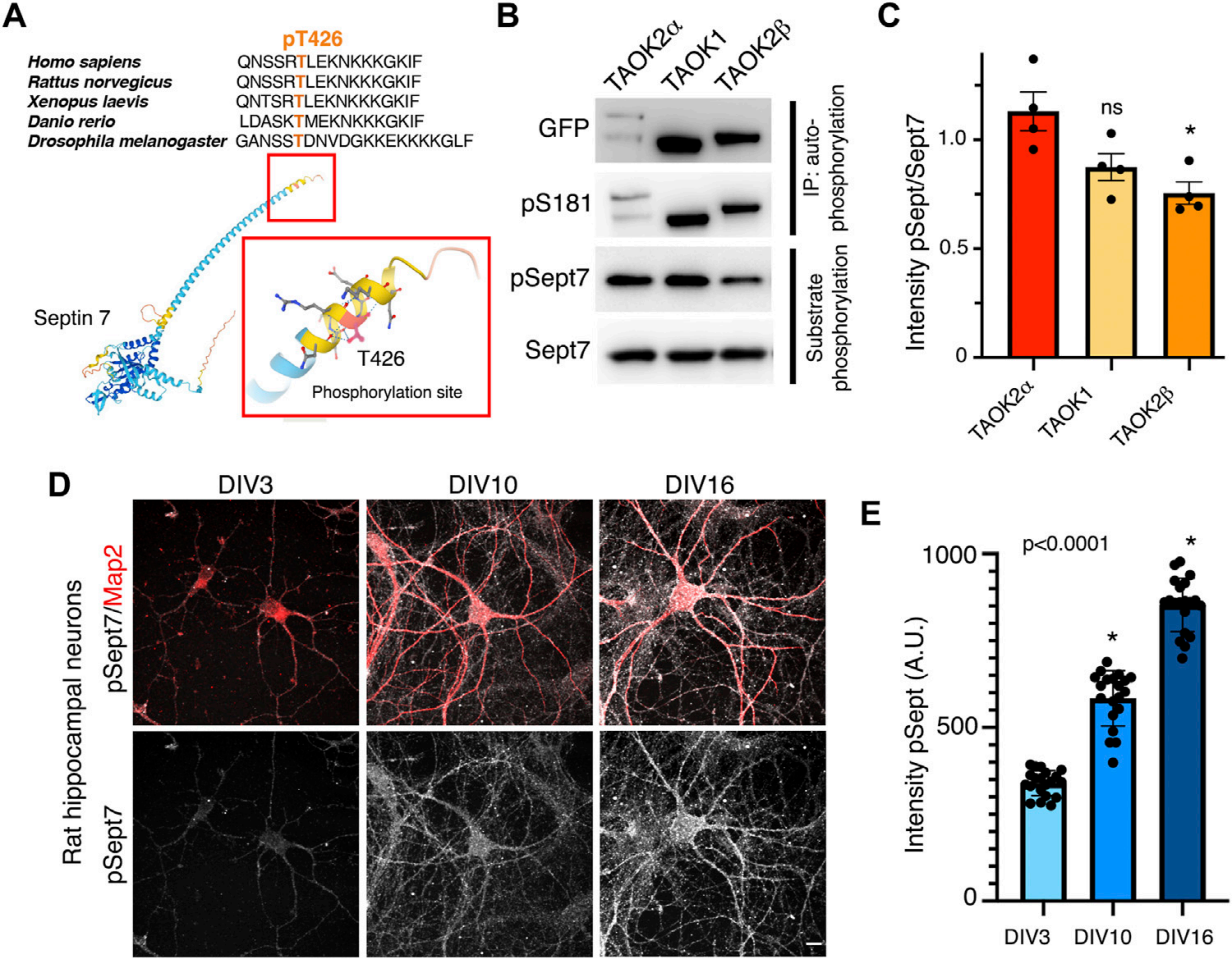
TAO kinases phosphorylate the C-terminal tail of Sept7 during neuronal development. **(A)** Top: multiple sequence alignment of the C-terminal tail of Sept7 demonstrating its evolutionarily conserved sequence including the phosphorylation site. The phosphorylated residue T426 is shown in orange. Bottom: AlphaFold2.0 predicted protein structure of Sept7. Magnified inset depicts the T426 phosphorylation site (red) in the C terminal tail of Sept7. **(B)** Western blot shows kinase activity of sfGFP-TAOK2α, sfGFP-TAOK1, or sfGFP-TAOK2β expressed in HEK293T cells, immunoprecipitated with GFP antibody, and incubated with purified GST-Sept7 C-terminal tail (321–438 amino acids) for an *in vitro* kinase reaction. Substrate phosphorylation was determined by probing for phosphorylated Sept7 using the pT426 antibody. Total Sept7 was detected by antibody against GST tag. TAO kinase autophosphorylation was determined by probing for phosphorylated S181 residue. **(C)** Quantification of protein band intensity of Western blot in **(B)**. Bar graph displays the ratio of phosphorylated Sept7 band intensity to GST band intensity for each TAO kinase construct in which no significant difference was observed between TAOK2α and TAOK1 kinases. Error bars represent standard error of mean from *n* = 4 experiments. **(D)** Rat hippocampal neurons at DIV3, 10, and 16 were fixed and immunostained for endogenous phosphorylated Sept7 T426 and dendritic protein Map2. Scale bar represents 10 μm. **(E)** Mean fluorescence intensity of phosphorylated Sept7 T426 at distinct stages of neuronal development DIV3, 10, and 16 are plotted. Error bars represent standard error of mean for *n* = 20 neurons from three different experiments. Dunnett’s multiple comparison shows significant changes in pSept7 levels between each stage.

Sept7 was identified through an unbiased chemical and genetic screening as a phosphorylation target of TAOK2 (Yadav et al., 2017). The site of phosphorylation residue T426 lies in the extended C-terminal coiled coil tail of Sept7. This threonine residue which harbors the consensus site for TAO kinases p[S/T]-X-X[R/H/K] is evolutionarily highly conserved (Figure 1A). Members of thousand-and-one amino acid kinase (TAOK) family contain a highly conserved N-terminal kinase domain, while their C-terminal domains considerably vary (Supplementary Figure S1A). TAO kinases, TAOK1 and spliced isoforms of TAOK2, α and β, are highly expressed in the brain (de Anda et al., 2012 and Allen Brain Atlas), and both TAOK1 and TAOK2 have been associated with neurodevelopmental disorders (Richter et al., 2018; Dulovic-Mahlow et al., 2019; van Woerden et al., 2021). TAOK2, both α and β, as well as TAOK1 have been shown to be important for several aspects of neuronal development including dendritic spine formation (Yasuda et al., 2007; Ultanir et al., 2014; Yadav et al., 2017; van Woerden et al., 2021). Recently, we discovered that TAOK2α is an endoplasmic reticulum–associated kinase with a transmembrane C-terminal region (Nourbakhsh et al., 2021). Given the structural conservation among the kinase domain of TAO kinases and their high expression in the brain (Hu et al., 2021), we tested whether closely related kinase TAOK1 and the alternatively spliced isoforms of TAOK2, TAOK2α, and TAOK2β could also phosphorylate Sept7 at T426. GFP-tagged TAOK1, TAOK2α, and TAOK2β were expressed independently in HEK293T cells, immuno-precipitated using GFP antibody and then incubated with purified GST-Septin7 protein (Supplementary Figure S1B) in an *in vitro* kinase reaction. Using a phosphospecific antibody (pT426) against the Sept7 T426 residue, we found that TAOK1, TAOK2α, and TAOK2β could each phosphorylate purified GST-Sept7 (Figures 1B,C). All of the immunoprecipitated TAO kinases were active as they could autophosphorylate themselves on the conserved residue S181 (TAOK1-2) in the kinase domain. Level of Sept7 phosphorylation by TAOK2α was significantly higher than that by TAOK2β, while phosphorylation by TAOK1 was not statistically different from TAOK2α. Furthermore, by immunostaining cultured hippocampal neurons at different stages of development, we found that phosphorylated Sept7 (T426) levels increased in neurons as it matured from DIV3 and DIV10 to DIV16 (Figures 1D,E). This is consistent with the increase in TAOK1, TAOK2α, and β expression in the mouse cortex from embryonic stages to the perinatal stage which then persists throughout adulthood (de Anda et al., 2012 and Allen Brain Atlas). These data suggest that the conserved family of TAO kinases show redundancy in Septin7 phosphorylation, where depending on the cellular context potentially multiple kinases of the TAO family can phosphorylate Sept7 C-terminal tail.

### Altered Dynamics of Phosphorylated Septin 7

**FIGURE 2:**
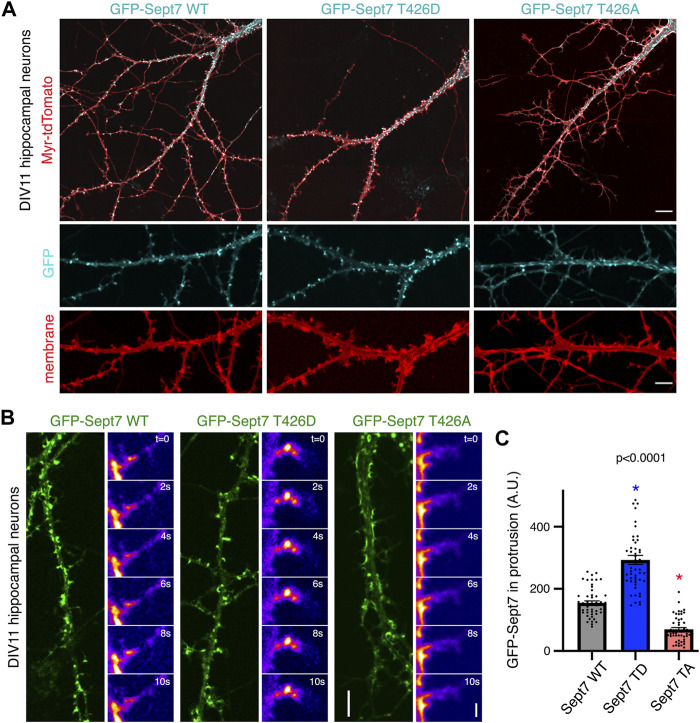
Phosphorylation of Sept7 alters its localization and dynamics. **(A)** Hippocampal neurons were transfected with GFP-tagged Sept7-WT, phosphomimetic Sept7-T426D, or phosphomutant Sept7-T426A along with membrane marker myristoylated-tdTomato at DIV9 and then fixed at DIV11. Phosphomimetic GFP-Sept7 (T426D) expression leads to early maturation of dendritic spines compared to Sept7-WT, while phosphomutant Sept7-T426A expressing neurons exhibited only filopodial protrusions. **(B)** Montage of time lapse images showing DIV11 hippocampal neurons transfected at DIV9 with GFP-tagged wild-type Sept7-WT, phosphomimetic Sept7-T426D, or phosphomutant Sept7-T426A. Phosphomimetic Sept7-T426D is stably localized within the dendritic spine head, phosphomutant Sept7-T426A localizes to the base of the dendritic spine, whereas the wild-type Sept7-WT shows dynamic changes in its localization within protrusions. Scale bar represents 5 µm (green) and 1 µm (montage), respectively. **(C)** Quantification of GFP intensity in protrusions of DIV11 neurons transfected with GFP-tagged wild-type Sept7-WT, phosphomimetic Sept7-T426D, and phosphomutant Sept7-T426A. Values represent mean intensity, error bars represent SEM and *n* = 50 protrusions from 10 neurons in each condition. Dunnett’s multiple comparison test, *p* < 0.0001.

We had previously reported that phosphorylated Sept7 is enriched in the dendritic spine head and is important for dendritic spine formation and stability of PSD95 (Yadav et al., 2017). Expression of Sept7 phosphomutant T426A leads to failure of dendritic spine maturation, resulting in exuberant dendritic filopodia and shaft synapses (Yadav et al., 2017). To test the role of Sept7 phosphorylation during neuronal maturation, we expressed wild-type (WT), phosphomimetic (T426D), and phosphomutant (T426A) Sept7 in DIV9 neurons and imaged them at DIV11, a developmental stage where filopodial protrusions have not yet developed into mature mushroom dendritic spines. We found that expression of GFP-tagged phosphomimetic Sept7 (T426D) as opposed to WT Sept7 led to early maturation of dendritic spines in hippocampal neurons (Figure 2A). In contrast, expression of phosphomutant Sept7 T426A as expected resulted in extensive filopodial protrusions (Figure 2A). We found that even at this early stage of development at DIV11, there was accumulation of GFP-Sept7 in dendritic spines in neurons expressing phosphomimetic Sept7 T426D. We performed confocal live imaging of neurons expressing either the wild-type (WT), phosphomimetic (T426D), or phosphomutant (T426A) GFP-tagged Sept7. Live imaging of dendritic protrusions at DIV11 revealed that wild-type GFP-Sept7 exhibited dynamic changes in its accumulation within the filopodial protrusions within 10 s time period. In contrast, phosphomimetic Sept7 (T426D) remained stably localized within the dendritic spine head, while the phosphomutant Sept7 (T426A) predominantly was localized at the base of the dendritic spine (Figure 2B, montage over 10 s). Quantification of Sept7 intensity in dendritic protrusions in DIV11 neurons revealed a significant increase in Sept7 T426D expressing neurons compared to WT, while there was a dramatic decrease in intensity of Sept7 T426A compared to WT (*n* = 50 protrusions, 12 neurons from three experiments, and one way ANOVA). Based on these data showing distinct localization and dynamics of Sept7 in its phosphorylated and unphosphorylated states at residue T426, we hypothesized that there must be unique interaction partners of phosphorylated Sept7 that modulate its discrete properties.

### Proteomic Identification of Septin 7 Phosphorylation-Dependent Binding Partners

**FIGURE 3:**
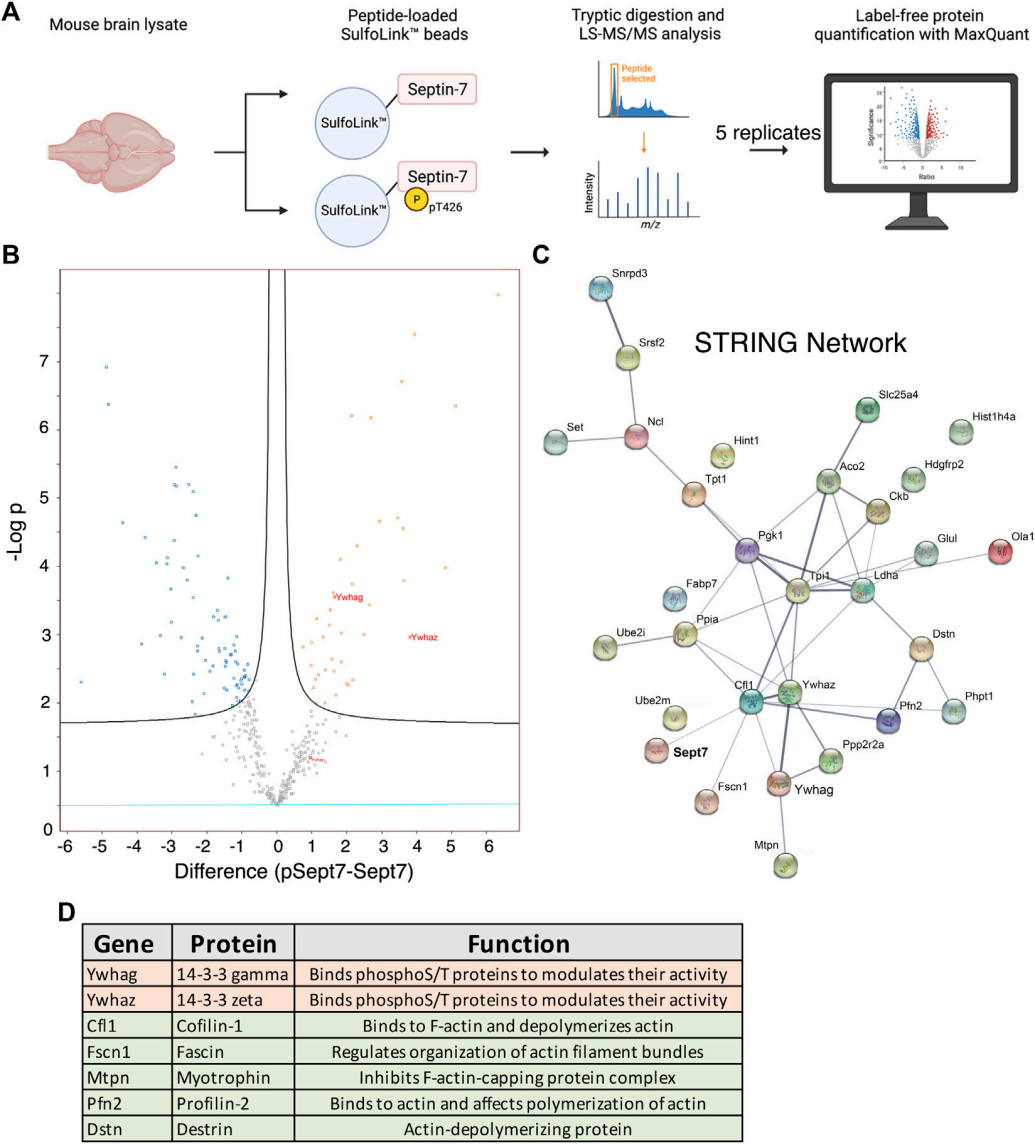
Proteomic identification of Septin 7 phosphorylation-dependent binding partners. **(A)** Workflow shows the proteomic strategy for identifying Sept7 phosphorylation-dependent binding partners. **(B)** Volcano plot displays the difference in association of proteins with phosphorylated (pT246) Sept7 versus unphosphorylated Sept7 based on five replicate pulldowns. 14-3-3 protein isoforms are labeled in red. **(C)** The protein–protein association network of candidate pSept7 interaction partners. The STRING network of identified proteins that significantly associate with phosphorylated Sept7 tail (pT426) versus non-phosphorylated Sept7 tail. Network nodes represent interacting proteins, and network edges indicate both functional and physical protein associations. Strength of the association is denoted by the thickness of the edges. **(D)** 14-3-3 family of proteins as well as several actin binding and depolymerizing proteins were found to significantly associate with Sept7 when phosphorylated at T426.

We set out to identify proteins that specifically interact with Sept7 following phosphorylation at residue T426 in its C-terminal tail. This was achieved using an unbiased proteomic strategy to pull down proteins from P15 mouse brain lysate with a synthetic peptide corresponding to Sept7 C-terminal tail residues 416–438 (Figure 3A). This peptide of 22 amino acids was unique to Sept7, as BLAST analyses did not identify any other protein harboring this sequence. Next, the phosphorylated (pSept7) and non-phosphorylated (Sept7) peptides were coupled to beads. Agarose beads activated with iodoacetamide were used for covalent immobilization of peptides corresponding to pSept7 and Sept7 peptides. Peptide coupled beads were then incubated with mouse brain lysate. Proteins that were bound to peptide beads were enzymatically digested and then subjected to mass spectrometry-based identification. Label free quantification was used to measure the relative abundance of proteins bound to Sept7 and pT426-Sept7 (Figure 3A). In our analyses of five replicates of peptide pulldowns, we found 33 proteins (Table 1) that were significantly associated with phosphorylated Septin 7 peptide over unphosphorylated peptide (Figure 3B). These proteins were analyzed by STRING11.2 and the most significant biological process was identified as regulation of cytoskeletal organization including actin-binding proteins as well as the phosphoprotein regulating proteins of the 14-3-3 family (Figures 3C,D). The 14-3-3 protein family is a group of highly conserved acidic proteins highly expressed in the mammalian brain (Boston et al., 1982; Ferl et al., 2002). In humans, there are seven known isoforms of 14-3-3: β, γ, ε, η, δ, τ, and ζ (Gardino et al., 2006). 14-3-3 proteins are ubiquitous regulatory proteins that form both homo- and heterodimers, and their dimerization provides binding sites for association with one or two phosphorylated ‘client’ proteins (Gardino et al., 2006; Uhart and Bustos, 2013). Among the candidate pSept7-binding partners identified by our mass spectrometry experiment (Figure 3C), we were specifically interested in 14-3-3 proteins as 1) binding of 14-3-3 proteins is largely determined by phosphorylation of the client, with the isoforms typically binding phosphorylated serine/threonine motifs (Aitken, 1996; Fu et al., 2000); 2) 14-3-3 are expressed in the brain and important for synapse development; and 3) binding of phosphoproteins to 14-3-3 can serve diverse purposes, including preventing protein–protein interactions, scaffolding, inducing conformational changes, protecting phosphorylation sites, and promoting or preventing ubiquitination.

**TABLE 1:** List of proteins identified by Mass Spectrometry that associate with Septin 7 when phosphorylated at residue T426.

Protein name	Gene	Function
14-3-3 protein gamma	Ywhag	Binds phospho S/T proteins and moduIates their of activity
14-3-3 protein zeta/delta	Ywhaz	Binds phospho S/T proteins and modulates their of activity
Myotrophin	Mtpn	Regulates growth of actin filaments. Inhibits the activity of the F-actin-capping protein complex
Destrin	Dstn	Actin-depolymerizing protein. Severs actin filaments (F-actin) and binds to actin monomers (G-actin)
Fascin	Fscnl	Organization of actin filament bundles and the formation of microspikes and stress fibers
Profilin-2	Pfn2	Binds to actin and affects the structure of the cytoskeleton
Cofilin-1	Cfll	Binds to F-actin and exhibits F-actin depolymerizing activity
Glutamine synthetase	Glul	Catalyzes the production ofglutamine and 4-am inobutanoate (gamma-aminobutyric acid, GABA)
Histidine triad nucleotide-binding protein 1	Hintl	Hydrolyzes purine nucleotide phosphoramidates
Triosephosphate isomerase	Tpil	Triosephosphate isomerase 1
Eukaryotic translation initiationfactor 5A-1; 5A-2	Eif5a; Eif5a2	mRNA-binding protein involved in translation elongation
Fatty acid-binding protein, brain	Fabp7	Belongs to the calycin superfamily
Translationally-controlled tumor protein	Tptl	Involved in calcium binding and microtubule stabilization
Heterogenous nuclear ribonucleo protein A3	Gm6793; Gm9242; Hnrnpa3	Plays a role in cytoplasmic trafficking of RNA
SUMO-conjugating enzyme UBC9	Ube2i	Necessary for sumoylation
Protein SET	Set	Multitasking protein, involved in apoptosis, transcription, and histone chaperoning
Guanine nucleotide-binding protein G(l)/G(S)/G(T) subunit beta-1; beta-2; beta-4	Gnb1; Gnb2; Gnb4	Guanine nucleotide-bind ing proteins act as a modulator in various transmembrane signaling events
NEDD8-conjugating enzyme Ubc12	Ube2m	Ubiquitin conjugating enzyme
Ubiquitin-conjugating enzyme E2 D2; Ubiquitin-conjugating enzyme E2 D3; D2B	Ube2d2; Ube2d3; Ube2d2b	Ubiquitin conjugating enzyme
Serine/threonine-protein phosphatase 2A 55 kDa regulatory subunit B alpha isoform	Ppp2r2a	Protein Phosphatase
Serine/arginine-rich splicing factor 2	Srsf2	Necessary for thesplicing of pre-mRNA
ADP/ATP translocase 1	Slc25a4	Involved in mitochondrial ADP/ATP transport
Peptidyl-prolyl cis-trans isomerase A; Peptidyl-prolyl cis-trans isomerase A	Ppia	PPlases accelerate the folding of proteins
14 kDa phosphohistidine phosphatase	Phptl	Exhibits phosphohistidine phosphatase activity
Hepatoma-derived growth factor-related protein 2	Hdgfrp2	Associates with chromatin
Histone H4	Histlh4a	Core component of nucleosome
Obg-like ATPase 1	Olal	Obg-like ATPase 1; Hydrolyzes ATP, and can also hydrolyze GTP with lower efficiency
Small nuclear ribonucleoprotein Sm D3	Snrpd3	plays an important role in the splicing of cellular pre-mRNAs
l-lactate dehydrogenase A chain	Ldha	Lactatedehydrogenase A
Phosphoglycerate kinase 1	Pgkl	Catalyzes one ofthe two ATP producing reactions in theglycolytic pathway
Aconitate hydratase, mitochondrial	Aco2	Catalyzes the isomerization of citrateto isocitrate *via* cis-aconitate
Creatine kinase B-type	Ckb	Reversibly catalyzes thetransfer of phosphate between ATP and various phosphogens
Nucleolin	Ncl	Induces chromatin decondensation by binding to histone H1

### 14-3-3 Proteins Associate with Septin 7

**FIGURE 4:**
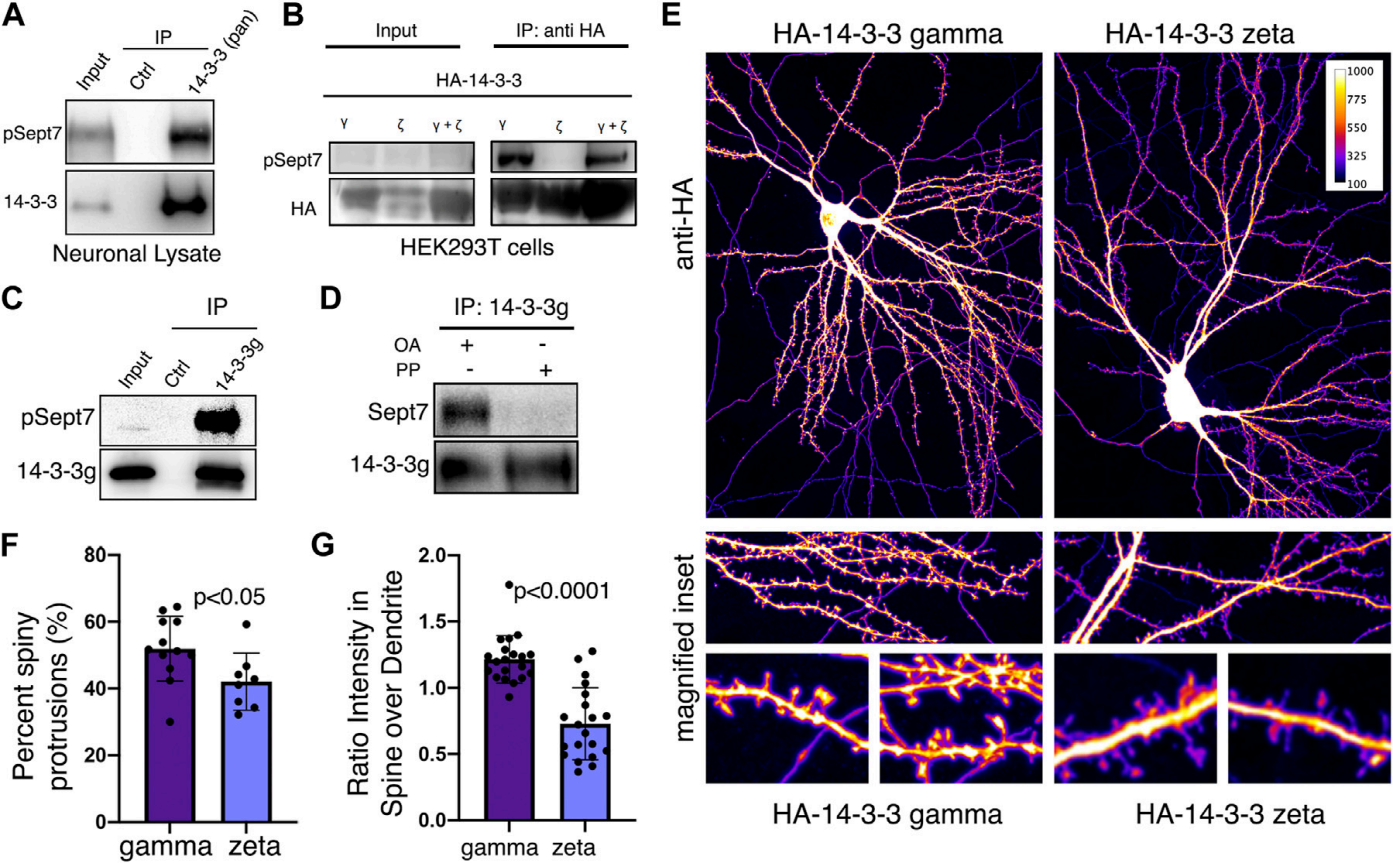
14-3-3γ associates with phosphorylated Sept7 and enhances dendritic spine maturation. **(A)** Western blot of endogenous 14-3-3 protein in DIV18 hippocampal neurons immunoprecipitated with 14-3-3 (pan) antibody and probed for 14-3-3 and phosphorylated Sept7 (pT426) (*n* = 3 experiments). **(B)** Western blot of HA-14-3-3γ, HA-14-3-3ζ, or both HA-tagged isoforms co-expressed along with GFP-Sept7 in HEK293T cells immunoprecipitated with HA antibody and probed for HA and phosphorylated Sept7 (pT426). Phosphorylated Sept7 co-immunoprecipitated with HA-14-3-3γ and HA-14-3-3(ζ+γ) but not HA-14-3-3ζ (*n* = 3 experiments). **(C)** Western blot shows coimmunoprecipitation of phosphorylated Sept7 with endogenous 14-3-3γ. Lysate from DIV18 hippocampal neurons immunoprecipitated with 14-3-3 (gamma) antibody and probed for 14-3-3γ and phosphorylated T426 Sept7 (*n* = 3 experiments). **(D)** Western blot shows phosphorylation dependent interaction of Sept7 with endogenous 14-3-3γ. DIV18 hippocampal neurons were treated with okadaic acid (OA) or DMSO, and lysate treated with or without lambda protein phosphatase (PP) were then immunoprecipitated with 14-3-3 (gamma) antibody and probed for 14-3-3γ and total Sept7 (*n* = 3 experiments). **(E)** Fixed images of DIV14 hippocampal neurons co-transfected with membrane marker myristoylated tdTomato and HA-14-3-3γ or HA-14-3-3ζ were immunostained for HA tag. Scale bar is 10µm, and image psuedocolored to represent fluorescence intensity. **(F)** Bar graph depicts percent of protrusions with mature, spiny morphology in DIV14 hippocampal neurons transfected with HA-14-3-3γ or HA-14-3-3ζ. HA-14-3-3γ expression results in significantly more spiny, mature protrusions (*p* < 0.05). Values indicate mean, error bars represent SEM and *n* > 8 neurons each. **(G)** Bar graph shows ratio of 14-3-3 fluorescent intensity in spines to intensity in corresponding dendritic shaft in DIV14 hippocampal neurons transfected with HA-14-3-3γ or HA-14-3-3ζ. Values indicate mean percent, error bars represent standard error of mean, and *n* = 20 spines from five neurons each.

We next validated whether 14-3-3 proteins were *bona fide* interacting proteins of Sept7 using co-immunoprecipitation assays. Using an antibody that recognizes all isoforms of 14-3-3 (pan 14-3-3 polyclonal), we immunoprecipitated 14-3-3 proteins from neuronal lysate obtained from DIV18 cultured embryonic rat hippocampal neurons and then probed for phosphorylated Sept7 using the pT426 Sept7 antibody. We found that indeed pSept7 coimmunoprecipitated with 14-3-3 proteins from neuronal lysates (Figure 4A, *n* = 3 experiments). Since 14-3-3 zeta (ζ) and gamma (γ) were the isoforms most enriched in our proteomic data (Figure 3D), and are also highly expressed in the brain (Berg et al., 2003), we next tested if there was an interaction of these isoforms with phosphorylated Sept7. We co-expressed HEK293T cells with GFP-Sept7 along with either HA-tagged 14-3-3 zeta, 14-3-3 γ, or both isoforms together. Using the HA antibody, we pulled down the 14-3-3 isoforms and then probed for phosphorylated Sept7 using the pT426 antibody. We found that pSept7 co-immunoprecipitated strongly with 14-3-3 γ and 14-3-3(ζ + γ) but not 14-3-3ζ (Figure 4B, *n* = 4 experiments). Furthermore, using a gamma isoform specific antibody, we found that endogenous 14-3-3γ from neuronal lysates co-immunoprecipitated pSept7 (Figure 4C, *n* = 3). Next, we tested whether the interaction between Sept7 and 14-3-3γ was dependent on phosphorylation. Neuronal lysates at DIV18 were either treated with lambda protein phosphatase or buffer and then incubated with immunoprecipitated 14-3-3γ. When immunoprecipitates were then probed for the presence of total Sept7, we found that while in untreated condition Sept7 bound 14-3-3γ, this interaction was not detected when lysate was treated with protein phosphatase (Figure 4D, *n* = 3). These data suggest that the interaction between Sept7 and 14-3-3γ is phosphorylation dependent. Next, to determine the effect of expression of different 14-3-3 isoforms on dendritic spine maturation, we co-expressed HA-tagged 14-3-3γ and 14-3-3ζ along with membrane marker myr-tdTomato in cultured DIV12 hippocampal neurons. Neurons were fixed at DIV14 and stained with anti-HA antibody to visualize the localization of overexpressed 14-3-3 proteins (Figure 4E). In neurons overexpressing HA-14-3-3γ, about 52% of protrusions were spiny mushroom mature spines, while 42% of protrusions were spiny in neurons expressing HA-14-3-3ζ (Figure 4F). Interestingly, HA-14-3-3γ was enriched in dendritic spines with a spine/dendrite ratio of 1.2, whereas HA-14-3-3ζ primarily localized to the dendritic shaft and was not enriched in the spine head (Figure 4G). In summary, we found that Sept7 interacts with 14-3-3γ in a phosphorylation-dependent manner. Furthermore, overexpression of 14-3-3γ but not the zeta isoform leads to its enrichment in the dendritic spine head and enhances spine maturation.

### Sept7 Association With 14-3-3 Protects its Phosphorylation at T426

**FIGURE 5:**
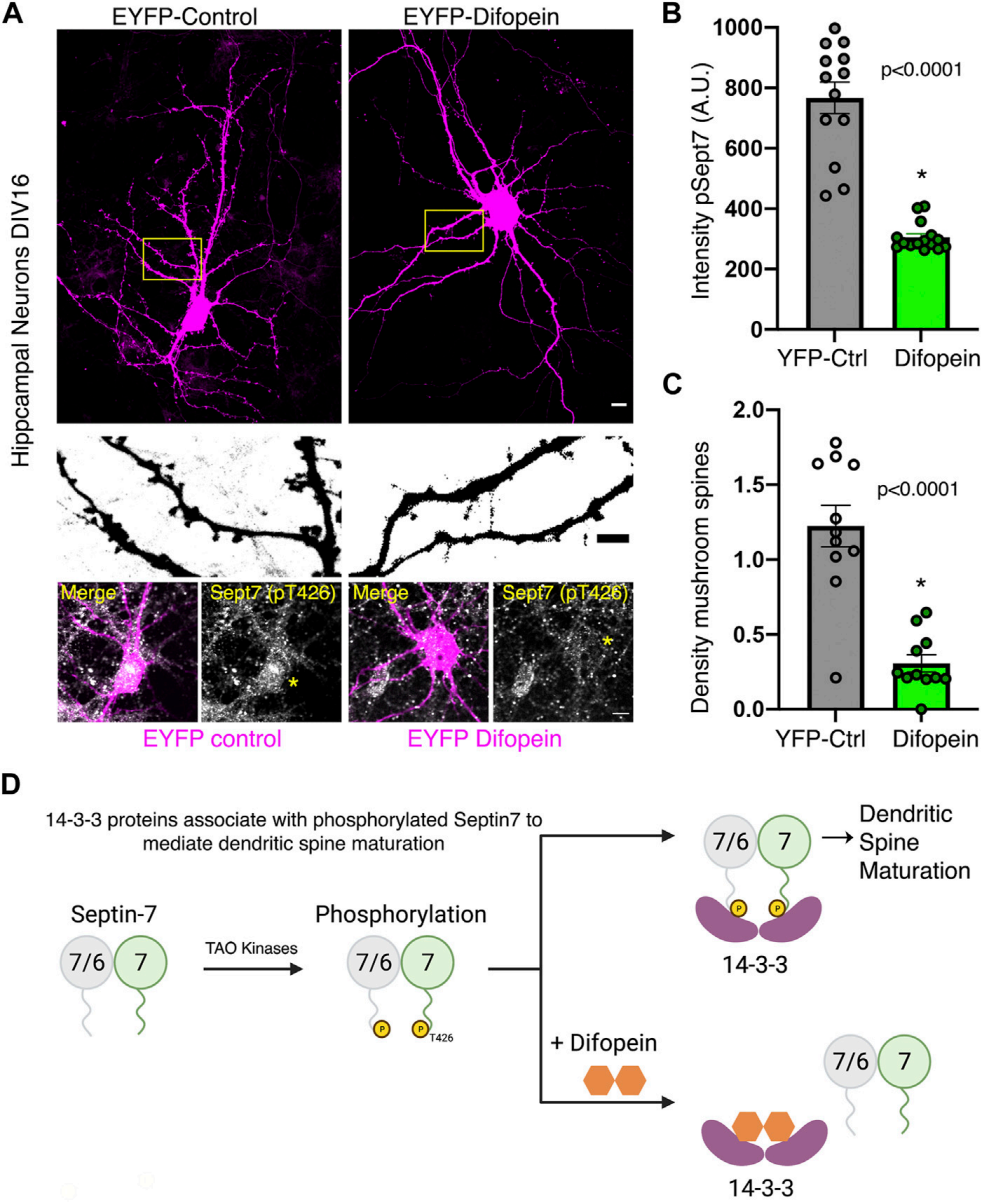
Association with 14-3-3 protects phosphorylation state of Sept7. **(A)** Fixed images of DIV16 hippocampal neurons transfected at DIV14 with EYFP-control or EYFP-Difopein (magenta), scale bar is 10 μm. Yellow inset is magnified to highlight dendritic protrusions shown in inverted grayscale, scale bar is 5 μm. Bottom row shows levels of pSept7 immunostaining (gray) in the neuronal soma of control and difopein-transfected (magenta) neurons. Transfected neurons are depicted by yellow asterisk, and scale bar is 5 μm. **(B)** Quantification of phosphorylated Sept7 levels in the soma of DIV16 hippocampal neurons expressing YFP-control or YFP-Difopein. YFP-Difopein expression results in significantly decreased levels of phosphorylated Sept7 (*p* < 0.0001). Values indicate mean; error bars represent SEM; and *n* = 13 and 15 neurons, respectively, from three distinct experiments. **(C)** Density of mushroom spiny protrusions in DIV16 hippocampal neurons expressing YFP-control or YFP-Difopein. Values indicate mean, error bars represent SEM, and *n* = 11 neurons each from three distinct experiments. **(D)** Schematic summarizes findings of this study showing 14-3-3 protein association modulates Sept7 function in neurons through protection of its phosphorylation state.

To test the functional consequence of 14-3-3 association with phosphorylated Sept7, we utilized a genetically expressed inhibitor of 14-3-3, EYFP-Difopein (dimeric 14-3-3 peptide inhibitor). This construct EYFP-Difopein is based on R18, a Raf-1–derived phosphopeptide, which is a high-affinity peptide antagonist of 14-3-3 proteins (Wang et al., 1999). Difopein is composed of two R18 coding sequences separated by a sequence coding for a short peptide linker in an EYFP fusion mammalian vector. Expressed Difopein is capable of disrupting 14-3-3/ligand binding (Masters and Fu, 2001). Difopein binds 14-3-3 without any isoform selectivity and thereby inhibits the interaction of all 14-3-3 isoforms with their physiological binding partners (Qiao et al., 2014). We transfected DIV14 hippocampal neurons with either control EYFP construct or EYFP-Difopein construct and then measured the levels of pSept7 in neurons fixed at DIV16 (Figure 5A). Quantification of pSept7 intensity in the soma of neurons expressing Difopein showed a significant decrease in the level of phosphorylated Sept7 compared to those expressing EYFP control, as detected by immunostaining using the pT426 Sept7 antibody (Figure 5A, yellow asterisk and 5B). Furthermore, neurons expressing Difopein had a dramatic decrease in density of mature mushroom spines compared to EYFP-Control transfected neurons (Figure 5A, middle row and 5C). These data suggest that interaction of the C-terminal tail of Sept7 with 14-3-3 proteins is protective of the phosphorylation status of Sept7 in neurons, revealing a mechanism for modulation of Sept7 function by association with 14-3-3 proteins (Figure 5D).

## Discussion

In this study, we utilize mass-spectrometry based discovery proteomics to identify binding partners that interact specifically with phosphorylated Sept7 in order to understand its role in dendritic spine development. Among the proteins we identified, we focus on the 14-3-3 family which are highly conserved phosphoprotein binding proteins that are important for brain development. We identify a specific interaction of pSept7 with 14-3-3γ (gamma) encoded by *YWHAG*. 14-3-3 gamma is highly expressed during brain development, and mainly in neurons. Our findings show that 14-3-3γ is specifically enriched in the dendritic spines, and that its expression in neurons leads to increased dendritic spine numbers.

14-3-3 proteins carry out a diverse array of functions in cellular processes, including in apoptosis (Masters and Fu, 2001), cell cycle progression (Peng et al., 1997), cytoskeletal rearrangements (Gohla and Bokoch, 2002), and neuronal growth (Cornell and Toyo-oka, 2017) that are mediated through the multitude of their phosphorylation dependent protein interactors. Accumulating evidence supports a profound role of 14-3-3 proteins in brain development. 14-3-3 proteins are important for neurogenesis, neuronal differentiation, and neuronal migration during cortical development (Cornell and Toyo-oka, 2017). Functional knockout of 14-3-3 proteins in mice brains with the dimeric 14-3-3 inhibitor Difopein results in reduced dendritic complexity and spine density accompanied by schizophrenia-related behaviors (Foote et al., 2015). 14-3-3 proteins are further required for hippocampal long term potentiation (LTP) and associative learning (Qiao et al., 2014), however, contribution of different 14-3-3 proteins in LTP was not ascertained. 14-3-3γ plays an important role in neuronal migration as either knockdown or overexpression of 14-3-3γ results in neuronal migration defects *in vivo* in mice (Wachi et al., 2015). Notably, 14-3-3γ expression is significantly reduced in human brain from Down Syndrome patients (Peyrl et al., 2002), indicating that perturbation of 14-3-3γ levels could contribute to disorders in human brain development. Furthermore, deletion of the *YWHAG* gene encoding 14-3-3γ is also associated with epilepsy and autistic traits in patients with atypical Williams Beuren syndrome due to deletions in 7q11.23 locus (Fusco et al., 2014).

Septins are important regulator of several aspects of neuronal development including dendrite growth, axon development and dendritic spine maturation. Our data show that phosphorylated Sept7 associates with 14-3-3 proteins. While our mass spectrometry data indicates that isoforms gamma and zeta bind specifically with phosphorylated Sept7 C-terminal tail, we only were able to biochemically validate the interaction with 14-3-3 gamma. It remains unknown whether different isoforms can heterodimerize to associate with phosphorylated Sept7. Using peptide inhibitor Difopein, we found that blocking interaction of Sept7 with 14-3-3 led to a decrease in the level of phosphorylated Sept7, highlighting the importance of the interaction. Since Difopein quenches the blocking site for phosphoproteins in all 14-3-3 proteins, our study did not directly test isoform specific effect of blocking Sept7 interaction with 14-3-3. Contribution of phosphorylated Sept7 and hence its protection by 14-3-3 proteins in these diverse neuronal contexts will be an important area of study. Dysfunction in both 14-3-3 and septins have been associated with various diseases including neurodegenerative diseases (Ide and Lewis, 2010) (Foote and Zhou, 2012) (Marttinen et al., 2015). In addition to the 14-3-3 proteins, several actin binding proteins were also identified in our proteomic study. Cofilin, an actin binding protein important for dendritic spine maturation was identified as a phosphorylation dependent Sept7 interacting protein (Gu et al., 2010; Pyronneau et al., 2017). Notably, phosphorylated cofilin also associates with 14-3-3 proteins (Gohla and Bokoch, 2002). Further understanding the interplay of septins, actin binding proteins, and 14-3-3 proteins in dendritic spine development may provide important insights into neurodevelopment and pathophysiology underlying neurological disorders.

## Data Availability

The original contributions presented in the study are included in the article/Supplementary Material, further inquiries can be directed to the corresponding author.
