# Supplementary figures and images for: Proteomic Identification of Phosphorylation-Dependent Septin 7 Interactors that Drive Dendritic Spine Formation
Source: Front Cell Dev Biol. 2022 May 4;10:836746. doi: 10.3389/fcell.2022.836746 (PMC9114808; doi:10.3389/fcell.2022.836746)

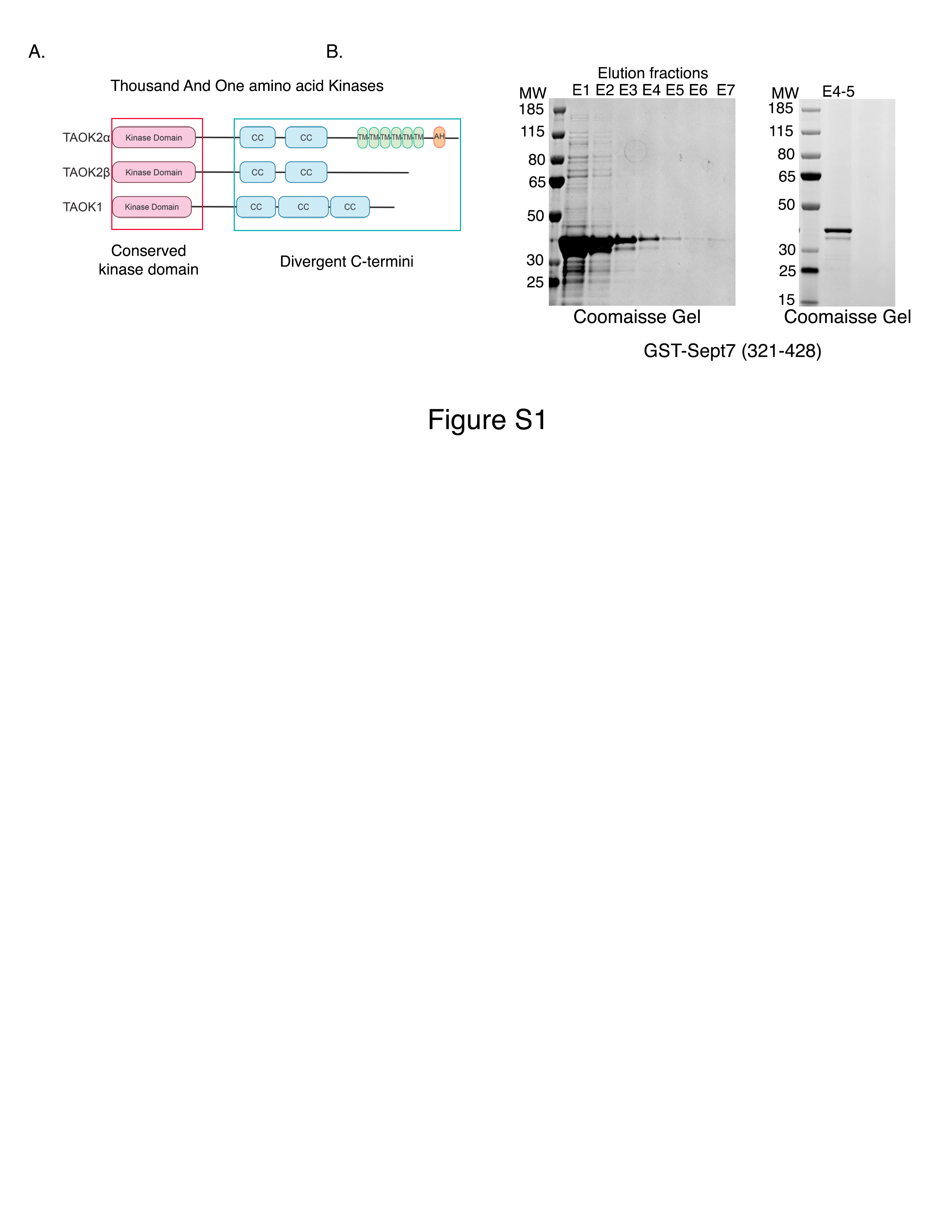

Supplement: Supplementary file 1 [file Image1.tif]
